# Bone Grafting in Scaphoid Avascular Necrosis: A Systematic Review Comparing Distal Radius and Iliac Crest Donor Sites

**DOI:** 10.7759/cureus.98944

**Published:** 2025-12-10

**Authors:** Jideofor Okoye, Ahmed A Ali, Shenouda R Shehata Abdelmesih, Shashwat Shetty, Akeem Balogun, Jarallah Alkhazendar, Ifrah Saleem

**Affiliations:** 1 Trauma and Orthopedics, Mersey and West Lancashire Teaching Hospitals NHS Trust, Rainhill, GBR; 2 Emergency Medicine, Mubarak Al-Kabeer Hospital, Jabriya, KWT; 3 Orthopedics and Traumatology, Royal Gwent Hospital, Newport, GBR; 4 Orthopedics, Hillingdon Hospital, Uxbridge, GBR; 5 Surgery, National Orthopaedic Hospital, Igbobi, Lagos, Lagos, NGA; 6 Trauma and Orthopedics, Lister Hospital, East and North Hertfordshire Teaching NHS Trust, Stevenage, GBR; 7 General Practice, Dow University of Health Sciences, Civil Hospital Karachi, Karachi, PAK

**Keywords:** avascular necrosis, distal radius, iliac crest, scaphoid nonunion, vascularized bone graft

## Abstract

Scaphoid nonunion with proximal pole avascular necrosis (AVN) presents a significant surgical challenge due to compromised vascularity and limited healing potential. Bone grafting has emerged as an effective strategy, with donor site selection, distal radius versus iliac crest, being crucial to clinical outcomes. This systematic review evaluated five studies, including 262 patients (distal radius vascularized bone grafting (DR-VBG): 53; iliac crest grafts: 209), comparing DR-VBG and iliac crest grafts in scaphoid nonunion with AVN. DR-VBG consistently demonstrated faster union (7.9-8.6 months), superior anatomical restoration, and lower donor-site morbidity compared with iliac crest grafts, which, despite providing robust structural support, were associated with higher pain, gait disturbances, nerve injury, and scarring. Functional outcomes were comparable when union was achieved; however, DR-VBG showed subtle advantages in carpal alignment and long-term wrist biomechanics. These findings support DR-VBG as the preferred donor site in AVN-complicated scaphoid nonunion, while iliac crest grafts remain a viable option when greater structural volume is required.

## Introduction and background

Fractures of the scaphoid are particularly challenging because the proximal pole relies on retrograde blood flow, and once nonunion occurs, especially with avascular necrosis (AVN) of the proximal fragment, progressive collapse, carpal malalignment, and arthrosis become significant concerns. The presence of AVN in scaphoid nonunion markedly reduces healing potential because the biological environment is compromised: studies show that conventional nonvascularized grafts achieve union rates of around 62% when AVN is present, compared with about 80% when it is absent. [1] Over the past decade, surgeons have therefore turned to vascularized bone grafting (VBG) in these difficult cases, aiming to provide both structural support and intrinsic vascular supply to the necrotic scaphoid segment [2].

The donor site for VBG is a critical decision. Local pedicled grafts harvested from the dorsal or volar aspect of the distal radius (e.g., 1,2-intercompartmental supraretinacular artery-based grafts) offer the advantages of anatomical proximity, minimal donor-site morbidity, and bone stock well suited in size and curvature to the scaphoid [3]. By contrast, the iliac crest donor site, long used for bone grafting, provides abundant corticocancellous bone and strong mechanical support, yet harvest is associated with higher pain levels, gait disturbance, sensory nerve injury, and more visible scarring [4]. When AVN complicates scaphoid nonunion, the need for enhanced biology via VBG becomes more compelling; thus, the comparison between distal radius and iliac crest donor grafts is not simply about union but about balancing biology, anatomy, and donor morbidity.

Biomechanically and biologically, VBG has been shown (in both animal and human studies) to accelerate revascularization, preserve osteocyte viability, support early mechanical load, and improve healing relative to non-vascularized grafts in AVN scenarios [5]. Anatomical restoration of scaphoid length, correction of humpback deformity, and normalization of carpal alignment are equally important contributors to functional outcome, beyond the achievement of union alone. In this context, donor site choice may influence not only healing time and success but also wrist motion, grip strength, pain, and long-term arthritis risk. Therefore, understanding outcomes of distal radius VBG (DR-VBG) versus iliac crest donor grafts in scaphoid nonunion with AVN is highly relevant for surgical decision-making.

The aim of this review is to compare vascularized bone grafts harvested from the distal radius with those harvested from the iliac crest in the treatment of scaphoid nonunion complicated by AVN. The primary aim is to compare union rate, time to union, functional outcomes (including wrist range of motion and grip strength), and complication/failure rates between the two donor-site options. The secondary aim is to evaluate donor-site morbidity (pain, sensory disturbance, and gait or functional limitation), anatomical restoration of scaphoid length and carpal alignment, and pathophysiological revascularization or osteogenic viability associated with each donor-site choice.

## Review

Materials and methods

Search Strategy

A comprehensive search strategy was developed according to the Preferred Reporting Items for Systematic reviews and Meta-Analyses (PRISMA) guidelines to ensure systematic identification of all relevant studies evaluating vascularized bone grafts from the distal radius and iliac crest for scaphoid nonunion with AVN [6]. Electronic databases, including PubMed, Embase, Scopus, and the Cochrane Library, were searched from their inception to October 2025. The search combined MeSH terms and keywords related to “scaphoid nonunion”, “avascular necrosis”, “vascularized bone graft”, “distal radius”, “iliac crest”, and “proximal pole”. Reference lists of included articles and relevant reviews were manually screened to identify additional eligible studies. No language restrictions were applied initially; however, non-English articles without accessible full texts were excluded during screening.

Eligibility Criteria

Eligibility criteria were framed using the Patient, Intervention, Comparison, Outcome (PICO) structure [7]. The population consisted of patients with scaphoid nonunion complicated by proximal pole AVN. The intervention included any type of vascularized bone graft harvested from the distal radius, while the comparator comprised nonvascularized or vascularized grafts from the iliac crest. Eligible outcomes included union rate, time to union, functional recovery, carpal alignment, donor-site morbidity, graft viability, and complication or failure rates. Studies were included if they were randomized trials, prospective or retrospective cohort studies, or comparative studies. Exclusion criteria included case reports, review articles, cadaveric or biomechanical studies, animal experiments, conference abstracts without full data, studies without clear AVN confirmation, or those evaluating non-scaphoid pathologies.

Study Selection

Titles and abstracts retrieved from the database search were screened independently by two reviewers to identify potentially eligible studies, ensuring consistency with the predefined PICO criteria. Full texts of shortlisted studies were then assessed for eligibility based on methodological clarity, AVN confirmation, population relevance, and reporting of outcomes of interest. Disagreements were resolved through consensus or adjudication by a third reviewer. Studies were only included if they directly compared distal radius vascularized grafts with iliac crest grafts or provided extractable outcome data relevant to the comparison.

Data Extraction

A standardized data extraction template was developed to collect key variables, including study design, authorship, sample size, patient demographics, AVN-confirmation method, type of graft used, fixation technique, and postoperative rehabilitation protocols. Extracted outcomes included union rates, time to radiological union, wrist range of motion, grip strength, pain scores, carpal alignment indices, donor-site morbidity, and complication or reoperation rates. Two reviewers performed the extraction independently, and data discrepancies were cross-checked against the original texts to maintain accuracy and reduce reporting bias. When required, corresponding authors were contacted for clarification or missing data.

Risk of Bias Assessment

Risk of bias for randomized trials was assessed using the Cochrane Risk of Bias tool (ROB-2) [8], evaluating sequence generation, allocation concealment, blinding, outcome reporting, and attrition management. Nonrandomized comparative studies and prospective cohorts were evaluated using the ROBINS-I tool to assess confounding, patient selection, intervention classification, deviations from intended interventions, missing data, and measurement bias [9]. Each study was assigned a rating of low, moderate, or serious risk of bias. Assessment was performed independently by two reviewers, and disagreements were resolved by consensus to ensure reliability and methodological transparency.

Data Synthesis

A qualitative synthesis was performed due to heterogeneity in study design, follow-up duration, fixation methods, and outcome reporting. Findings were narratively integrated, focusing on union rates, healing time, functional and anatomical restoration, graft biology, and donor-site morbidity associated with each technique. Where possible, trends were compared across studies to identify consistent advantages or limitations of distal radius versus iliac crest donor sites. The synthesis adhered to PRISMA recommendations and emphasized clinical relevance, biomechanical plausibility, and long-term functional implications rather than simple pooled effect estimates, which were not feasible due to variability in available data.

Results

Study Selection Process

**Figure 1:**
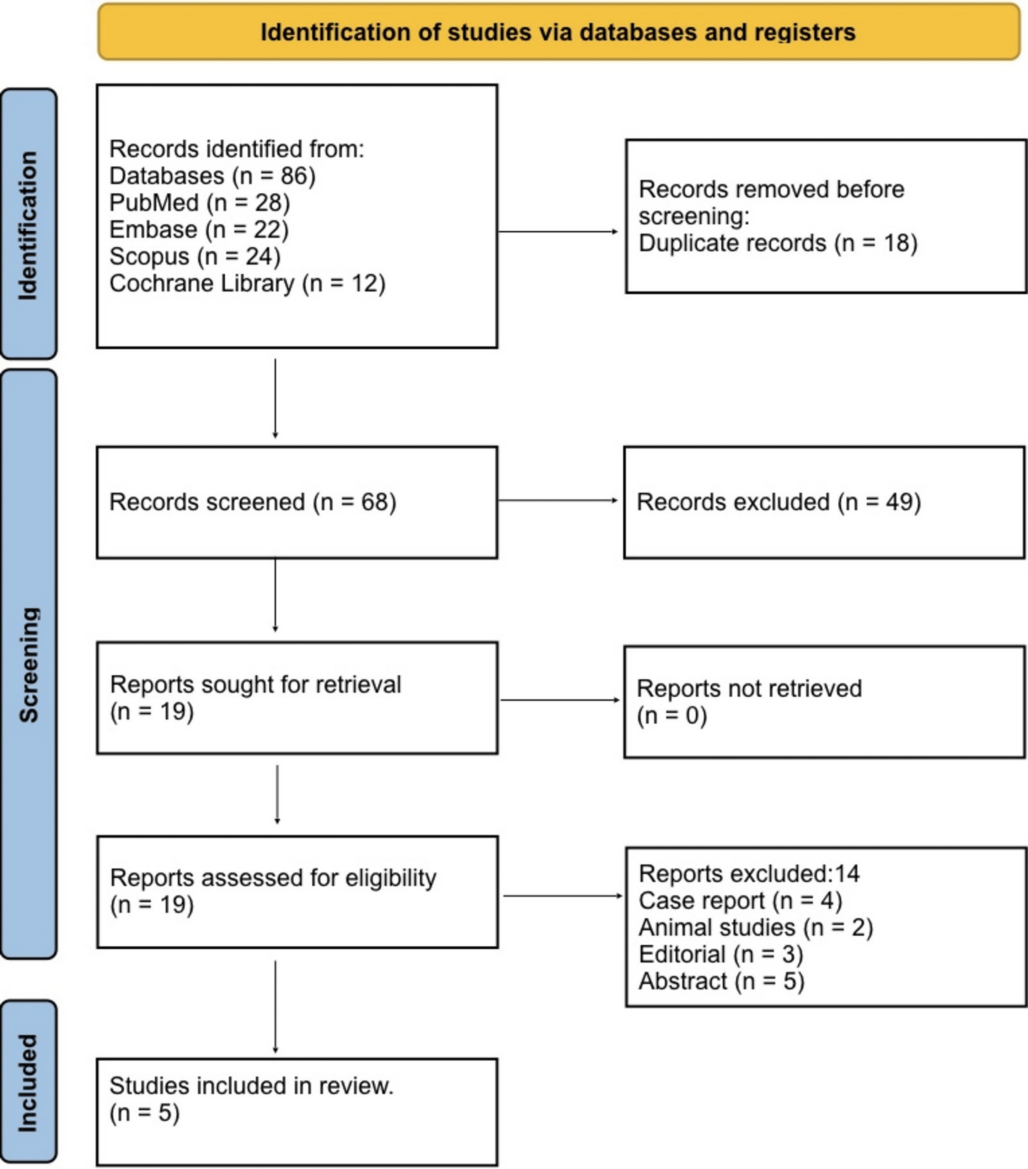
PRISMA 2020 flow diagram of the study selection process PRISMA, Preferred Reporting Items for Systematic reviews and Meta-Analyses

Figure 1 shows the PRISMA flow diagram of study selection. A total of 86 records were identified through database searching, including PubMed (n = 28), Embase (n = 22), Scopus (n = 24), and the Cochrane Library (n = 12). After the removal of 18 duplicates, 68 records remained for title and abstract screening. At this stage, 49 records were excluded because they did not meet the predefined eligibility criteria. The remaining 19 full-text articles were assessed for eligibility. Of these, 14 were excluded for reasons including case reports (n = 4), animal studies (n = 2), editorials or narrative reviews (n = 3), and conference abstracts without full data (n = 5). Ultimately, five studies met all inclusion criteria and were included in the final systematic review.

Characteristics of the Selected Studies

Table 1 illustrates that DR-VBG demonstrates faster union and lower donor-site morbidity compared with iliac crest grafts. Braga-Silva et al. reported quicker union (7.97 vs. 8.89 months), improved biological viability in AVN cases, and minimal donor-site pain, although three failures were noted in the VBG group [10]. Garg et al. observed no major difference in union or functional outcomes between distal radius and iliac crest nonvascularized grafts; however, iliac crest grafts were associated with significantly higher pain (Visual Analogue Scale 7.1 vs. 4.2) [11]. Smeraglia et al. demonstrated higher union rates with DR-VBG (88% vs. 75%) and faster healing (8.6 vs. 11.7 weeks), along with reduced morbidity compared with iliac crest harvest [12]. Harpf et al. showed that free vascularized iliac crest grafts can achieve high union rates (≈91.7%) but are associated with considerable donor-site morbidity, including lateral femoral cutaneous nerve (LFCN) neuropraxia and pelvic deformity [13]. Maraşlı et al. found DR-VBG provided the highest union rate (100%), optimal anatomical restoration, and the lowest morbidity when compared with medial femoral condyle or iliac crest grafts [14].

**Table 1:** Characteristics of the selected studies AVN, avascular necrosis; DR-VBG, distal radius vascularized bone graft; IC-NVBG, iliac crest nonvascularized bone graft; ICSRA, intercompartmental supraretinacular artery; LFCN, lateral femoral cutaneous nerve; MFC-VBG, medial femoral condyle vascularized bone graft; NVBG, nonvascularized bone graft; VAS, Visual Analogue Scale; VBG, vascularized bone graft

Author and year	Population (P)	Exposure/Intervention (I)	Comparator (C)	Outcomes (O)	Pathophysiological findings	Anatomical impact	Donor-site morbidity (comparison)	Complications	Failure rate
Braga-Silva et al. (2008) [10]	80 scaphoid nonunions	Distal radius vascularized bone graft (1,2-ICSRA)	Iliac crest nonvascularized graft	Time to union: 7.97 months (DR-VBG) vs. 8.89 months (IC-NVBG). Functional outcome similar	VBG improves biological viability in proximal pole AVN	Maintains carpal alignment; restores scaphoid length	Iliac crest: high pain; DR-VBG: minimal morbidity	Three failures in the VBG group	VBG: 3/35, IC: 0/45
Garg et al. (2013) [11]	100 randomized patients	Distal radius local nonvascularized bone graft	Iliac crest nonvascularized bone graft	Union rate and functional outcome not significantly different	Similar biological incorporation	DR graft matches local anatomy	Iliac crest pain higher (VAS 7.1 vs. 4.2)	Wrist stiffness (DR), wound pain (IC)	Failures rare; no significant difference
Smeraglia et al. (2020) [12]	21 total: DR-VBG (n = 9) vs. IC-NVBG (n = 12)	Volar distal radius vascularized bone graft	Iliac crest nonvascularized graft	Union: 88% (DR-VBG) vs. 75% (IC-NVBG). Time to union: 8.6 weeks vs. 11.7 weeks	VBG accelerates revascularization in AVN	Better correction of scaphoid length with VBG	Iliac crest morbidity higher (gait pain, scar)	1 minor wound issue (IC group)	VBG: 1/9, IC: 3/12
Harpf et al. (2001) [13]	60 scaphoid reconstructions	Free vascularized iliac crest bone graft	No DR comparison (used as standard comparator)	Union ≈91.7%, good functional recovery	Strong osteogenic potential from periosteal circulation	Structural support for proximal pole collapse	High morbidity: LFCN neuropraxia (31%), scar issues (40%), donor deformity (63%)	Wound issues, nerve irritation	~8.3%
Maraşlı et al. (2021) [14]	IC-NVBG (8), MFC-VBG (7), DR-VBG (9)	DR-VBG (1,2-ICSRA) and medial femoral condyle graft	Iliac crest nonvascularized graft	Union: 100% (DR-VBG), 85.7% (MFC), 87.5% (IC-NVBG). Functional scores similar	VBG provides superior revascularization for proximal pole necrosis	DR-VBG restores carpal height, stable fixation	DR-VBG least painful; IC worst morbidity	No major complications in DR-VBG	IC: 1/8, MFC: 1/7, DR-VBG: 0/9

Risk of Bias Assessment

The overall risk of bias across the included studies ranged from low to serious, largely influenced by study design and methodological limitations. The randomized controlled trial (RCT) by Garg et al. demonstrated a low risk of bias, with adequate randomization, allocation concealment, and blinded outcome assessment, although the sample size remained modest [11]. In contrast, the prospective cohort by Braga-Silva et al. showed a moderate risk, primarily due to the lack of randomization, despite having clear inclusion criteria and complete follow-up [10]. Similarly, the prospective comparative studies by Smeraglia et al. and Maraşlı et al. were rated as moderate risk of bias because of small sample sizes and nonrandomized designs, although both employed standardized outcome measures and transparent methodologies [12,14]. The case series by Harpf et al. was rated as having a serious risk of bias, as the absence of a comparator group and potential selection bias limited internal validity, despite clearly reported outcomes, as shown in Table 2 [13].

**Table 2:** Risk of bias assessment RCT, randomized controlled trial; ROBINS-I, Risk of Bias in Nonrandomized Studies of Interventions; RoB-2, Cochrane Risk of Bias tool for randomized trials

Study	Study design	Risk of bias tool	Risk of bias rating	Justification
Braga-Silva et al. (2008) [10]	Prospective cohort	ROBINS-I	Moderate	Single-center study, no randomization, but clear inclusion/exclusion criteria and complete follow-up
Garg et al. (2013) [11]	RCT	RoB-2	Low	Randomized, allocation concealment reported, outcome assessors blinded; minor risk due to small sample size
Smeraglia et al. (2020) [12]	Prospective comparative study	ROBINS-I	Moderate	Nonrandomized, but prospective design with clear outcome measures
Harpf et al. (2001) [13]	Case series (prospective)	ROBINS-I	Serious	No comparator group, potential selection bias, small sample; outcomes clearly reported
Maraşlı et al. (2021) [14]	Prospective comparative study	ROBINS-I	Moderate	Small groups, nonrandomized, but with clear inclusion/exclusion criteria, follow-up, and standardized outcome measures

Discussion

The management of scaphoid nonunion complicated by proximal pole AVN remains one of the most challenging scenarios in hand surgery. The compromised vascularity of the proximal fragment significantly impairs healing, making conventional nonvascularized grafts less reliable, with union rates reportedly around 62% in the presence of AVN compared to 80% in its absence [1]. This systematic review highlights that VBG, particularly using the DR-VBG, offers both biological and anatomical advantages over traditional iliac crest grafts (IC-NVBG or IC-VBG), supporting the premise that donor-site selection is critical for optimizing outcomes in these complex cases.

Across the included studies, DR-VBG consistently demonstrated faster union times, superior anatomical restoration, and lower donor-site morbidity. Braga-Silva et al. reported that DR-VBG achieved union in 7.97 months compared with 8.89 months for iliac crest nonvascularized grafts, with minimal donor-site discomfort, whereas iliac crest harvest was associated with higher pain and occasional gait disturbances [10]. Similarly, Smeraglia et al. showed that volar DR-VBG led to an 88% union rate versus 75% with IC-NVBG and shortened time to union (8.6 vs. 11.7 weeks), suggesting enhanced revascularization and preservation of osteocyte viability in the DR-VBG group [12]. These findings underscore the biological superiority of local vascularized grafts in delivering an intrinsic blood supply, which is critical for revascularizing necrotic proximal fragments.

Anatomical considerations further favor distal radius grafts. The DR-VBG matches the scaphoid curvature, preserves carpal height, and facilitates restoration of humpback deformity and carpal alignment more reliably than iliac crest grafts [12,14]. In contrast, iliac crest grafts, although offering abundant corticocancellous bone and robust mechanical support, frequently resulted in donor-site complications such as LFCN neuropraxia, gait disturbances, prominent scarring, and local pain, as noted by Harpf et al. [13]. These complications may impact postoperative rehabilitation and patient satisfaction, highlighting the importance of weighing structural benefits against morbidity.

Functionally, wrist motion and grip strength outcomes were generally comparable between the two donor sites when union was achieved [10,14]. However, subtle advantages in anatomical restoration with DR-VBG may translate into improved long-term wrist biomechanics and reduced risk of secondary degenerative changes. The slightly higher failure rates reported in some DR-VBG series (e.g., 3/35 in Braga-Silva et al.) likely reflect technical challenges in graft harvest, fixation precision, and patient selection rather than an inherent limitation of the donor site [10].

The limitations of the current evidence must be acknowledged. Most studies were small, single-center, and predominantly nonrandomized, introducing selection and reporting biases. Heterogeneity in graft type, fixation techniques, follow-up duration, and outcome assessment precluded meta-analysis and reduced the generalizability of findings. Furthermore, the lack of standardized measures for donor-site morbidity, AVN confirmation, and long-term functional outcomes limits the ability to draw definitive conclusions. Only one RCT was included, emphasizing the need for more rigorous research.

Future research should prioritize multicenter RCTs with adequate sample sizes comparing DR-VBG and iliac crest grafts. Standardized outcome metrics, including patient-reported outcomes, pain scales, carpal alignment indices, and long-term functional and radiological follow-up, will enhance evidence quality. Additional investigations into the role of adjunctive biological therapies, preoperative imaging for AVN assessment, and surgical technique optimization may further improve outcomes. Evaluating cost-effectiveness, learning curve, and quality-of-life impact could also guide donor-site selection in clinical practice.

## Conclusions

In the management of scaphoid nonunion with proximal pole AVN, DR-VBG consistently demonstrates faster union, superior anatomical restoration, and lower donor-site morbidity compared to iliac crest grafts. While iliac crest grafts provide robust structural support, their higher rates of pain, nerve injury, gait disturbance, and scarring make them less favorable as a first-line donor site. DR-VBG offers an optimal balance of biological enhancement, anatomical fidelity, and functional recovery, positioning it as the preferred reconstructive option in contemporary scaphoid surgery. Careful patient selection and surgical expertise remain critical, and further multicenter randomized studies are needed to strengthen the evidence and guide donor-site selection.
